# Device-assessed physical activity and sleep quality of post-COVID patients undergoing a rehabilitation program

**DOI:** 10.1186/s13102-024-00909-2

**Published:** 2024-05-29

**Authors:** Iris Poppele, Marcel Ottiger, Michael Stegbauer, Torsten Schlesinger, Katrin Müller

**Affiliations:** 1 Institute of Human Movement Science and Health, Faculty of Behavioral and Social Sciences, Chemnitz University of Technology, 09107 Chemnitz, Germany; 2 BG Hospital for Occupational Disease Bad Reichenhall, 83435 Bad Reichenhall, Germany

**Keywords:** post-COVID, Inpatient rehabilitation, Physical activity, Sleep quality, Accelerometry

## Abstract

**Background:**

An infection with SARS-CoV-2 can lead to persistent symptoms more than three months after the acute infection and has also an impact on patients’ physical activity behaviour and sleep quality. There is evidence, that inpatient post-COVID rehabilitation can improve physical capacity and mental health impairments, but less is known about the change in physical behaviour and sleep quality.

**Methods:**

This longitudinal observational study used accelerometery to assess the level of physical activity and sleep quality before and after an inpatient rehabilitation program. The study sample consists of 100 post-COVID patients who acquired COVID-19 in the workplace. Group differences related to sex, age, COVID-19 severity, and pre-existing diseases were also analysed.

**Results:**

Level of physical activity and sleep quality didn’t increase after rehabilitation. Overall, there is a high extent of inactivity time and poor sleep quality at both measurement points. Regarding group differences, male patients showed a significantly higher inactivity time before rehabilitation, and younger patients (< 55 years) spend significant more time in vigorous physical activity than older patients. Post-COVID patients with pre-existing cardiovascular, respiratory, and metabolic disease show slightly less physical activity than post-COVID patients without these comorbidities. Female patients and younger patients showed better sleep quality in some sleep parameters at both measurement points. However, no differences could be detected related to COVID-19 severity.

**Conclusions:**

Ongoing strategies should be implemented to address the high amount of inactivity time and the poor sleep quality in post-COVID patients.

**Supplementary Information:**

The online version contains supplementary material available at 10.1186/s13102-024-00909-2.

## Introduction

An infection with SARS-CoV-2 (severe acute respiratory syndrome coronavirus type 2) can manifest in a broad symptom range and even after several months after acute infection, many patients are experiencing physical, mental, and cognitive health impairments [1–4]. According to national and international Guidelines, we define post-COVID within this study as persistent symptoms (> 12 weeks) due to COVID-19 (coronavirus disease 2019), which cannot be explained by an alternative diagnosis [5, 6]. The prevalence of post-COVID is estimated to 6% according to population-based cohort studies [7–10]. The symptom cluster of post-COVID is very divers, including exercise intolerance, fatigue, sleeping disorders and mental and cognitive deficits. They can persist more than 24 months after acute COVID-19 and affect the patients’ quality of life and ability to work [1, 2, 11, 12]. 

During the restrictions of the COVID-19 pandemic, the general level of physical activity (PA) was reduced [13–15]. Accordingly, previous studies have reported an increased risk of getting infected with SARS-CoV-2, hospitalization and mortality due to COVID-19, and post-COVID when persons had a physical inactive lifestyle [16–21]. Therefore, physical inactivity represents a significant risk factor for developing long-term symptoms (post-COVID). Galluzzo, et al. [18] compared the reported PA level before and after COVID-19 and showed that almost 25% of participants stopped practicing PA after getting infected. Their results are in line with results of the study by Delbressine, et al. [22]. The mentioned studies assessed the amount of PA by questionnaires. Even if this measurement procedure is time and cost effective, the given subjective answers by the participants are often over estimated [23, 24], and can be influenced by cognitive impairments due to post-COVID [25]. There are only some studies, analysing the PA behaviour of post-COVID patients in a more objective manner with accelerometers (e.g., ActiGraph, GENEActiv, activPAL™), which should be favoured as a measurement method for valid recording of PA in terms of scope and intensity [26, 27].

Benitez, et al. [28] used accelerometery to assess the PA of post-COVID patients. The results indicated that patients spend Median (Mdn) = 34 min/day in moderate to vigorous physical activity (MVPA) and were reaching the WHO (World Health Organisation) PA recommendations (150 min MVPA/week) [29]. Plekhanova, et al. [30] reported a lower amount of MVPA below the recommended PA level. The female and male patients spend mean (M) = 12.1 to 12.6 h/day in an inactive state, which is in line with findings by van Bakel, et al. [31]. The authors of the mentioned studies compared their results with these of healthy population studies and stated, that post-COVID patients are less physical active, and thus, PA should be promoted within this patient cluster. There is strong evidence, that regular PA is linked to several health benefits e.g., improving the cardiovascular system, enhancing cognitive functions, and the reduction of depressive symptoms and anxiety [32]. Regarding chronic inflammation processes, which is one potential mechanism of post-COVID pathogenesis [33], PA can contribute to the attenuation of the inflammatory response and strengthen the muscle functional capacity. Sustained inactivity, on the other hand, has the opposite (negative) effects on inflammation and functional capacity [34–37].

The German S1 guideline Long-/Post-COVID refers to the PA recommendations of the WHO [29] as an important preventive and rehabilitative treatment strategy for post-COVID patients [5]. Rütten and Pfeifer [29] had already specified these PA recommendations in their “National guidelines for physical activity and the promotion of physical activity” for adults with chronic illnesses without contraindications to implement PA. So far, rehabilitation studies could verify that a multidisciplinary post-COVID rehabilitation program could improve physical function in post-COVID patients [38–41]. However, there are no studies available examining the effect of rehabilitation on the PA level of post-COVID patients. Studies with COPD patients could show that an improvement in physical function and exercise performance after rehabilitation is not automatically accompanied by an increase in PA in daily life [42, 43]. The study of Carl, et al. [44] with COPD patients could reveal, that the Physical Activity-related Health Competence predicts the level of PA (step counts), and thus, the competencies should be addressed within rehabilitation programs. A recent systematic review indicated that online interventions to increase PA can be effective if they go beyond simply transferring knowledge, e.g. by providing pedometers or video tutorials [45]. This will be particularly relevant for the development and implementation of aftercare strategies.

A systematic review revealed that the people infected with SARS-CoV-2 showed a prevalence of sleep disturbances of 52,4% [46] even several months after hospital discharge [47]. Sleep disturbances are characterised as problems falling or staying asleep and are mostly leading to daytime fatigue [48]. Even after 2 years of COVID-19 the prevalence is still high with 31%. The proportion was significantly higher than in a matched non-COVID-19 control group [49]. Obesity, female sex, duration of hospital stay, and mental health concerns are identified risk factors of experiencing sleep disturbances due to COVID-19 [28, 47, 48].

Most of the studies assessed sleep quality by questionnaires like the Pittsburgh Sleep Quality Index [50] or the Insomnia Severity Index [51]. Device based measurements of sleep characteristics of post-COVID patients such as the usage of accelerometers are rare. Plekhanova, et al. [30] and van Bakel, et al. [31] assessed with accelerometers the PA and sleep behaviour of post-COVID patients admitted to the hospital during the acute phase of infection. Patients with a better recovery process had better sleep parameters and the sleep duration per day was higher [30]. Compared to a control group, the post-COVID patients had significantly lower rates in sleep duration, regularity and efficiency indicating an impaired sleep quality [30, 52]. This finding could be confirmed by a study using polysomnography, the gold standard when assessing patients sleep [53]. According to Benitez, et al. [28] post-COVID patients had substantial more sleep periods during the day and wake up more often during the night.

Because of the bidirectional association between sleep quality and immune response [54], there is reason to focus on the treatment of sleep disturbances for post-COVID recovery [55]. Particularly, the inflammatory storm triggered by COVID-19 is able to cause changes in circadian rhythm and lead to impaired sleep [48, 54]. Vice versa sleep deprivation severely impairs the immune system functionality by disturbing the immune homeostasis [54, 56]. Poorer sleep quality causes longer recovery durations and an increased need for ICU (Intensive care unit) care after COVID-19 [57]. Further, mental disorders (e.g., depression, anxiety, PTSD), organic diseases (e.g., cardiovascular and respiratory diseases) and chronic pain can be also associated with sleep disturbances [58–61].

Except for post-COVID patients with a severe course of ME/CFS (myalgic encephalomyelitis/chronic fatigue syndrome) [62, 63], PA and physical exercise can contribute to an enhanced sleep quality [15, 64]. Thus, the implementation of an appropriate rehabilitation program will play a crucial role in the improvement of patients sleep and the recovery process of post-COVID. There are only a few studies investigating the effect of rehabilitation on sleep quality of patients with COVID-19 [16, 65]. Liu, et al. [65] revealed that patients after mild COVID-19 showed significant better sleep scores after pulmonary rehabilitation than the control group. The case-report of Young [16] describes several post-COVID cases suffering from different sleep disturbances (e.g., interruption of sleep, sleep latency, sleep efficiency). In all four analysed cases, specialised rehabilitation programs could reduce the symptoms of sleep disturbances after seven to ten weeks of rehabilitation.

In sum, the PA level of post-COVID patients is lower and sleep disturbances are confirmed even two years after the acute infection with SARS-CoV-2. To date, less is known about the impact of a post-COVID rehabilitation program on objective measured PA and sleep quality. The aim of the current study is to bridge this gap and to investigate the objectively measured PA and sleep quality of patients who acquired COVID-19 at the workplace at the beginning and their potential changes at the end of an inpatient post-COVID rehabilitation. Specifically, the current study will address the following three research questions:

1. What habitual PA behaviour and sleep quality do post-COVID patients show at the beginning of inpatient rehabilitation?
2. What changes in habitual PA behaviour and sleep quality of post-COVID patients occur after the course of inpatient rehabilitation?
3. What influence do sex, age, COVID-19 severity, and comorbidities have on habitual PA behaviour and sleep quality of post-COVID patients and their potential changes?

## Methods

This study was conducted at the Chemnitz University of Technology, Germany, in cooperation with the BG Hospital Bad Reichenhall. It is registered in the German Clinical Trials Register (DRKS) under DRKS 00022928. The study was approved by the Ethics Committee of the Bavarian State Medical Association (number 21,092) and the Ethics Committee of Chemnitz University of Technology (TU Chemnitz, Chemnitz, Germany), Faculty of Behavioural and Social Sciences (number V-427-17-KM-COVID-19-18022021).

### Study design and participants

This current study reports data from post-COVID patients from two measurement points before (T1) and after (T2) inpatient rehabilitation. It has to be mentioned, that this study is embedded in a longitudinal research project that collects data from further measurement points until 12 months after rehabilitation. The patients were recruited at the BG Hospital Bad Reichenhall after their respective accident insurance providers registered them for rehabilitation. When the patients in the post-acute phase of COVID-19 as a recognized occupational disease or work-related accident met the inclusion criteria (the patient is in the post-acute phase without evidence of infectivity, COVID-19 is recognised as occupational disease or work-related accident, confirmed ability to undergo rehabilitation, voluntary study participation) and did not meet the exclusion criteria (severe cardiological, internist, neurological, psychological and musculoskeletal diseases that were already present before COVID-19), they signed a written informed consent form. The current study presents the results of the first two measurement points before (T1) and after (T2) the inpatient rehabilitation period.

All the included patients went through an inpatient multidisciplinary post-COVID rehabilitation program at the BG Hospital Bad Reichenhall with a mean duration of 28.84 ± 5.16 days. In addition to medical treatment and care, patients participated in comprehensive physical and psychological treatments by specialists. For detailed information on the components of inpatient rehabilitation, see Müller, et al. [66].

**Fig. 1:**
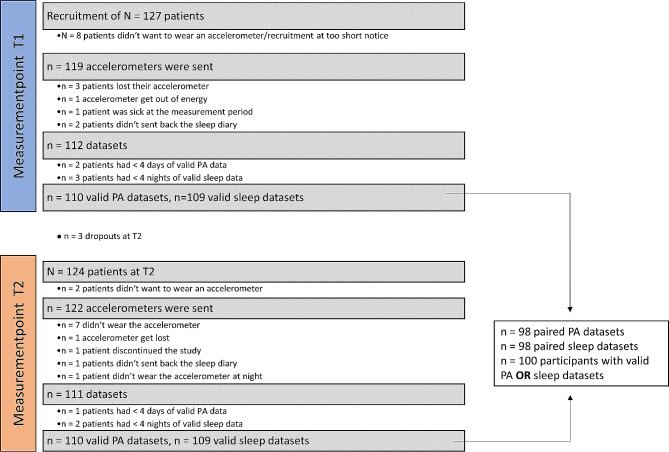
Flow chart of included PA and sleep datasets

At T1, out of 127 recruited patients 119 patients were sent an accelerometer (ActiGraph GT9X Link) with a corresponding sleep diary in form of a table for each wearing day, where they filled in when they went to bed, got up in the morning and to log non-wear periods (e.g., while taking a shower). Three patients lost their accelerometer, one accelerometer get out of energy, one patient was sick at the measurement period and two patients didn’t send back the sleep diary. Two patients didn’t have enough valid wearing time for the PA data (valid: ≥16 h/day [67]; midnight-midnight) and three didn’t have enough valid time during the night (valid: ≥16 h/day [67]; noon-noon). Overall, there are 110 valid datasets at T1 for PA and 109 for the sleep analysis. At T1, there is M = 3.77% (± 4.65) non-wear time within the dataset. At T2, there were three dropouts (reasons: one patient reinfected with SARS-CoV-2, two patients were no longer interested in study participation) and two patients didn’t want to wear the accelerometer. Therefore, 122 accelerometers were sent at T2 regardless of whether the patients received an accelerometer or had a valid wearing time at T1. At T2, seven patients didn’t wear the accelerometer, one patient lost the accelerometer, one patient discontinued the study, one patient didn’t send back the sleep diary and one patient didn’t wear the accelerometer at night. Further, one patient didn’t have enough valid PA time and two patients didn’t have enough valid time during the night. In summary, there are 110 valid datasets for PA and 109 valid datasets for sleep analysis at T2. Non-wear time at T2 is M = 3.85% (± 5.12). In the present study, only data of the paired sample (PA: *N* = 98, sleep quality: *N* = 98) will be reported, since there is no significant difference between the excluded (unpaired) participants (PA: *n* = 12, sleep: *n* = 11) and the paired sample (Fig. 1).

The present study sample for the analyses includes in total 100 participants (73 females/ 27 males; Age: M = 51.13 ± 10.82 years) with valid datasets for PA and/or sleep quality (96 patients with valid PA and sleep data, 2 patients with valid PA data, 2 patients with valid sleep data). 65 patients were working within the healthcare sector (e.g., nurses, doctors, physiotherapists) whereas 35 are non-healthcare workers (e.g., administrative staff, industrial-/building technicians, social education staff, and teachers). During their acute stage of COVID-19, 71 patients experienced a mild to moderate course of disease, and 29 a severe to critical course of disease according to the WHO classification [68]. Within the study sample, 87 (87%) patients are overweight (BMI > 25 kg/m^2^). At T1, 8 (8%) patients were smokers, 39 (39%) patients were former smokers, and 53 (53%) patients did never smoke (Table 1).

**Table 1:** Characteristics of the study population

	*N* = 100^1^
Sex	
Male	27 (27%)
Female	73 (73%)
Age [years]	51.13 (10.82)
BMI [kg/m²]	31.36 (6.09)
Normal	13 (13%)
Overweight	34 (34%)
Obesity class I	29 (29%)
Obesity class II	16 (16%)
Obesity class III	8 (8%)
Smoking status	
Currently (every day)	4 (4%)
Currently (occasional)	4 (4%)
Former	39 (39%)
Never	53 (53%)
COVID-19 severity	
Mild-moderate	71 (71%)
Severe	24 (24%)
Critical	5 (5%)
Rehabilitation duration [days]	28.88 (5.16)
Interval COVID-19 - Rehabilitation [days]	402.45 (141.64)
Comorbidities prior to COVID-19	
Cardiovascular disease	53 (53%)
Respiratory disease	42 (42%)
Mental Illness	19 (19%)
Metabolic disease	64 (64%)

^1^M (SD); *n* (%)

### Sociodemographic data, anamnesis and post-COVID Symptoms

Sociodemographic data and post-COVID symptoms were obtained via post-delivered questionnaires. These were self-generated according to the current German COVID-19 and post-COVID guidelines [5] and the German Health Interview and Examination Survey for Adults [69, 70]. A semi-structured interview by a physician during anamnesis complemented the data from the questionnaires. Pre-existing comorbidities were obtained through the subscale of the work ability index [71].

### Accelerometery

To assess patients’ PA and sleep quality before and after inpatient rehabilitation, the patients were asked to wear an accelerometer (ActiGraph GT9X Link) on their right waist in their domestic environment. Mora-Gonzalez, et al. [72] reported a good accuracy for the accelerometer device worn on the waist with a mean absolute percentage error of 4.1% in normal gait speed. The data collection periods were at least seven days, for the first time two weeks before the rehabilitation program and for the second time two weeks after the rehabilitation programme. The accelerometer was initialized with the ActiLife 6 software (ActiGraph, Pensacola, FL, USA) to record triaxial accelerations with a sample frequency of 60 Hz. The patients received the wearable device by post together with the corresponding sleep diary. The patients had to wear the accelerometer 24 h per day with exception during swimming or having a shower. They were also instructed to keep the sleep diary. After one week, the patients send back the device with a prepaid envelope. The raw data was exported by the software ActiLife 6 to.gt3x files. The analysis of the raw accelerometer data was conducted with R Studio (2023.06.0) and the R-package GGIR (Version: 2.8-2) [67, 73, 74].

Participants data was excluded when they had less than 4 days of valid data (valid: ≥16 h/day) [75]. The GGIR package does autocalibrate the data by using local gravity as a reference value [76]. The acceleration metric ENMO (Euclidean Norm Minus One with negative values rounded to zero) was used and calculates the average magnitude of dynamic acceleration of the three axes corrected for gravity. To reduce the amount of data, the accelerometer values (unit: milligravitational (mg)) were averaged over 5s epochs. Further, there is a detection of non-wear periods. Within this study the default setting was used, which imputed invalid data (non-wear) by the average at similar time-points on different days of the week.

PA was categorised into four intensity levels according to WHO classification. For this purpose, the aggregated 5s epochs were classified into four activity intensities by using the following threshold values (unit: mg), which were validated by Hildebrand, et al. [77] using ergo-spirometry: inactive (< 47.4 mg, < 1.5 metabolic equivalent of task (MET)), light (< 69.1 mg, ≥ 1.5 MET), moderate (< 258.7 mg, ≥ 3 MET) and vigorous (≥ 258.7 mg, ≥ 6 MET) [77]. To assess the number of patients reaching the WHO guidelines of 150 min MVPA per week for a heathy lifestyle, MVPA was calculated by adding up the time participants spend in moderate and vigorous PA.

Sleep characteristics were also calculated by GGIR using the Cole-Kripke algorithm (based on the zero-crossing method) [78] and the sleep diary of the participants. First, the main sleep period is identified with the help of the sleep diary. Second, the Cole-Kripke algorithm, with a time threshold of 5 min is applied. If the recorded accelerometer values are below the threshold value for light PA during this period of time, this period is categorized as sleep. Following sleep variables were derived: time in bed (in hours; total time spend in bed according to the sleep diary), sleep duration (in hours; total sleep time during the time in bed), wake after sleep onset (WASO) (in hours; total time being awake after falling asleep the first time), sleep regularity (in %, “percentage probability of an individual being in the same state (asleep vs. awake) at any two time-points 24 h apart, averaged across the study” [79]), sleep efficiency (%; calculated by dividing the sleep duration by time in bed), sleep latency (time between going to bed and sleep onset). According to the current literature and international guidelines a sleep duration of six to 10 h during the night, a sleep efficiency above 85%, a sleep latency shorter than 30 min and being awake less than 51 min after sleep onset is considered as good sleep quality [80–83].

### Statistical analysis

All statistical tests were performed using R software (Version 4.2.1). To compare the variables before and after rehabilitation, the Wilcoxon singed-rank test was used since most of the variables were not normally distributed. The Mann-Whitney U test was conducted to detect possible group differences relating to sex (male (*n* = 27) vs. female (*n* = 73)), age (younger than 55 (*n* = 46) vs. at least 55 years (*n* = 54)), and COVID-19 severity (mild-moderate (*n* = 71) vs. severe-critical (*n* = 29)). The Mann-Whitney U-test was also used to compare group differences over time (Difference = T2-T1). Furthermore, pre-existing conditions (cardiovascular disease, respiratory disease, mental illness, metabolic disease) were considered and group differences were also calculated. The prevalence of dichotomous variables (self-reported post-COVID symptoms, reaching the WHO activity recommendations) at T1 and T2 was compared by using the McNemar test. To identify possible interaction effects between the above-mentioned groups a two-way ANOVA was conducted for each of the PA and sleep parameters at T1 and T2. The level of significance was set at *p* < 0.05. Effects sizes were reported as r. According to Fritz, et al. [84], an effect size r of 0.1 represents a ‘small’ effect size, 0.3 a ‘medium’ effect size, and 0.5 a ‘large’ effect size.

## Results

### Post-COVID symptoms

**Fig. 2:**
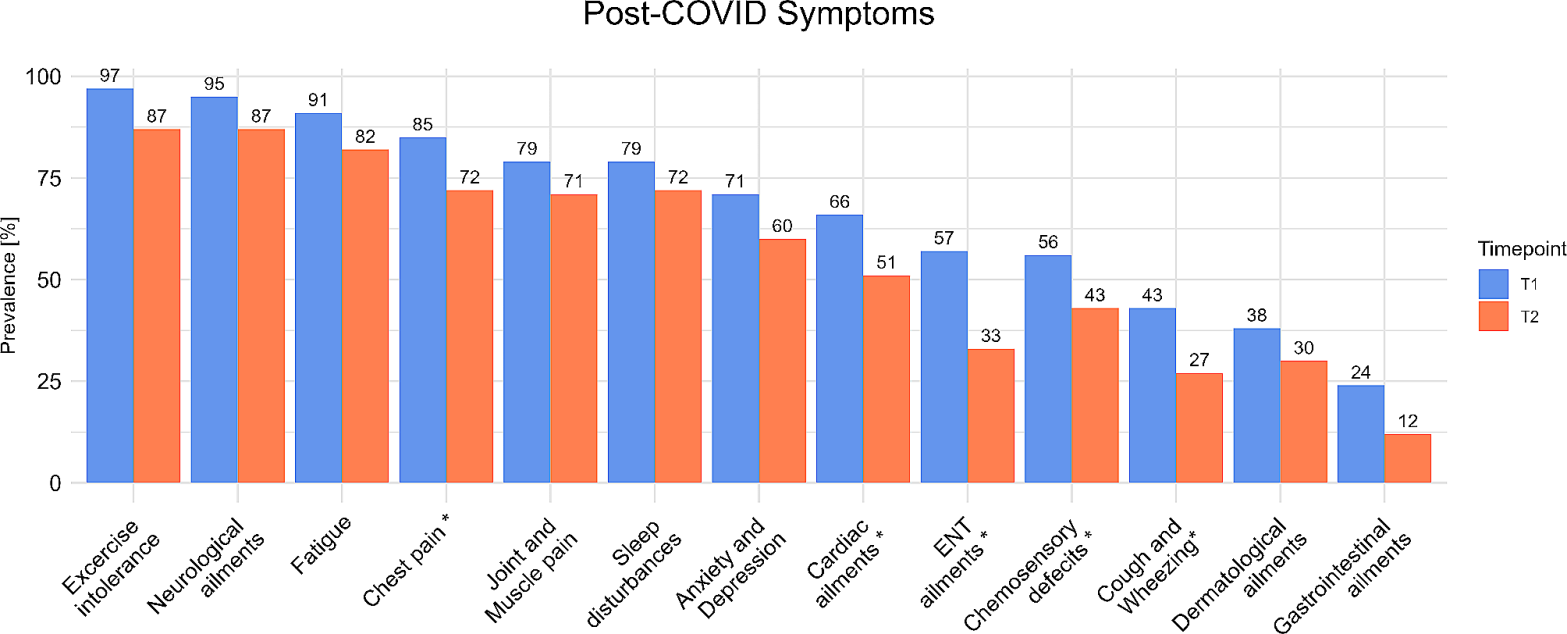
Symptom clusters of post-COVID patients before (T1, blue) and after (T2, orange) rehabilitation. * = Significant difference (*p* < 0.05). ENT = ear-nose-throat

Figure 2 illustrates 13 summarized symptom clusters according to Bahmer, et al. [4]. At the beginning of rehabilitation almost all patients are showing symptoms of exercise intolerance (97%), neurological ailments (95%) and fatigue (91%). Chest pain, Joint and Muscle pain, and sleep disturbances (85%, 79%, 79% respectively) are also very common among the observed post-COVID symptoms at T1. Descriptively, the prevalence of all observed symptoms decreased after rehabilitation. The results of the McNemar test did show a significant decrease in the prevalence in five symptoms (*p* < 0.05): chest pain decreased from 85 to 72%, cardiac ailments from 66 to 51%, ear-nose-throat (ENT) ailments from 57 to 33%, chemosensory deficits from 56 to 43%, and the prevalence from Coughing and Wheezing decreased from 43 to 27%. None of the observed symptoms increased in their prevalence after the rehabilitation program.

### Physical activity

**Fig. 3:**
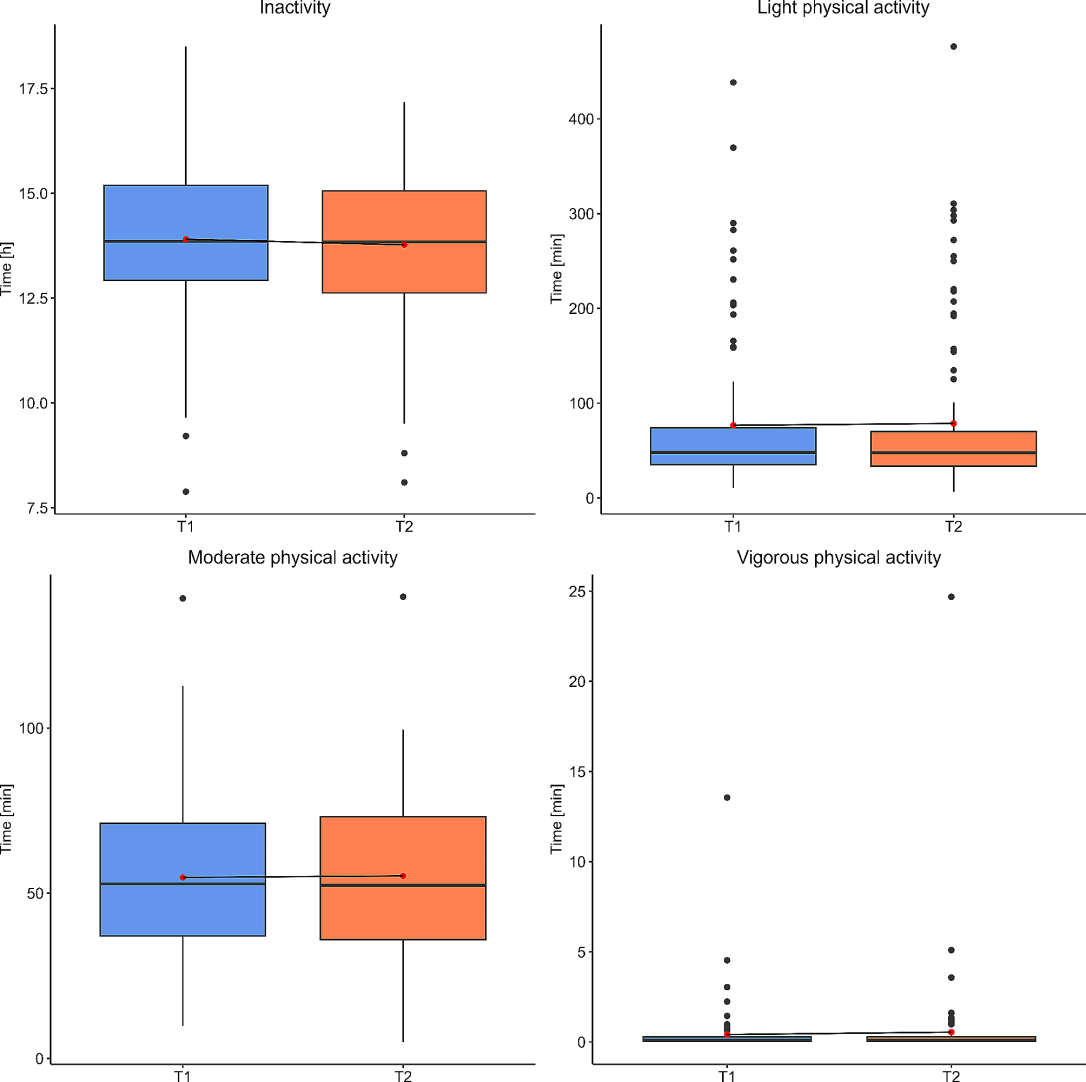
Distribution of the device assessed PA parameters of post-COVID patients before (T1, blue) and after (T2, orange) inpatient rehabilitation. The median score is displayed as solid black line within the boxplot. The mean value is represented as red point

At T1, the patients were Mdn = 13.91 h (Interquartile range (IQR): 12.96–15.10) inactive, Mdn = 47.11 min (IQR: 34.31–74.10) in light PA, Mdn = 51.19 min (IQR: 36.58–71.12) in moderate PA, and Mdn = 0.11 min (IQR: 0.04–0.31) in vigorous PA per day. Even if there was no significant difference in (in)activity levels after the rehabilitation program (*p* > 0.05) (Table 2; Fig. 3), the time spending inactive decreased slightly to Mdn = 13.88 h (IQR: 12.65–15.05) per day and 51 (52%) patients reduced their inactivity time after rehabilitation. At T2, the three PA intensity levels increased slightly over time. Patients were Mdn = 46.43 min (IQR: 32.78–69.65) lightly active, Mdn = 53.81 min (IQR: 35.98–72.87) moderately active and Mdn = 0.11 min (IQR:0.05–0.34) intensively active. Within the current study sample, 92 (93.9%) patients are reaching the activity goal according to WHO recommendation at T1 and 94 (95.9%) patients at T2. The McNemar’s chi-squared test with continuity correction didn’t reveal a significant difference between the two measurement timepoints (*p* > 0.05).

**Table 2:** PA of post-COVID patients before (T1) and after (T2) inpatient rehabilitation program

	Timepoint		**z**	**p**	**r**
	T1	T2			
	Median (IQR)	Median (IQR)			
Inactivity [h]	13.91 (12.96, 15.10)	13.88 (12.65, 15.05)	0.331	0.780	0.033
Light Activity [min]	47.11 (34.31, 74.10)	46.43 (32.78, 69.65)	0.257	0.788	0.026
Moderate Activity [min]	51.19 (36.58, 71.12)	53.81 (35.98, 72.87)	0.036	0.675	0.004
Vigorous Activity [min]	0.11 (0.04, 0.31)	0.11 (0.05, 0.34)	-0.210	0.789	-0.021

### Sleep quality

**Fig. 4:**
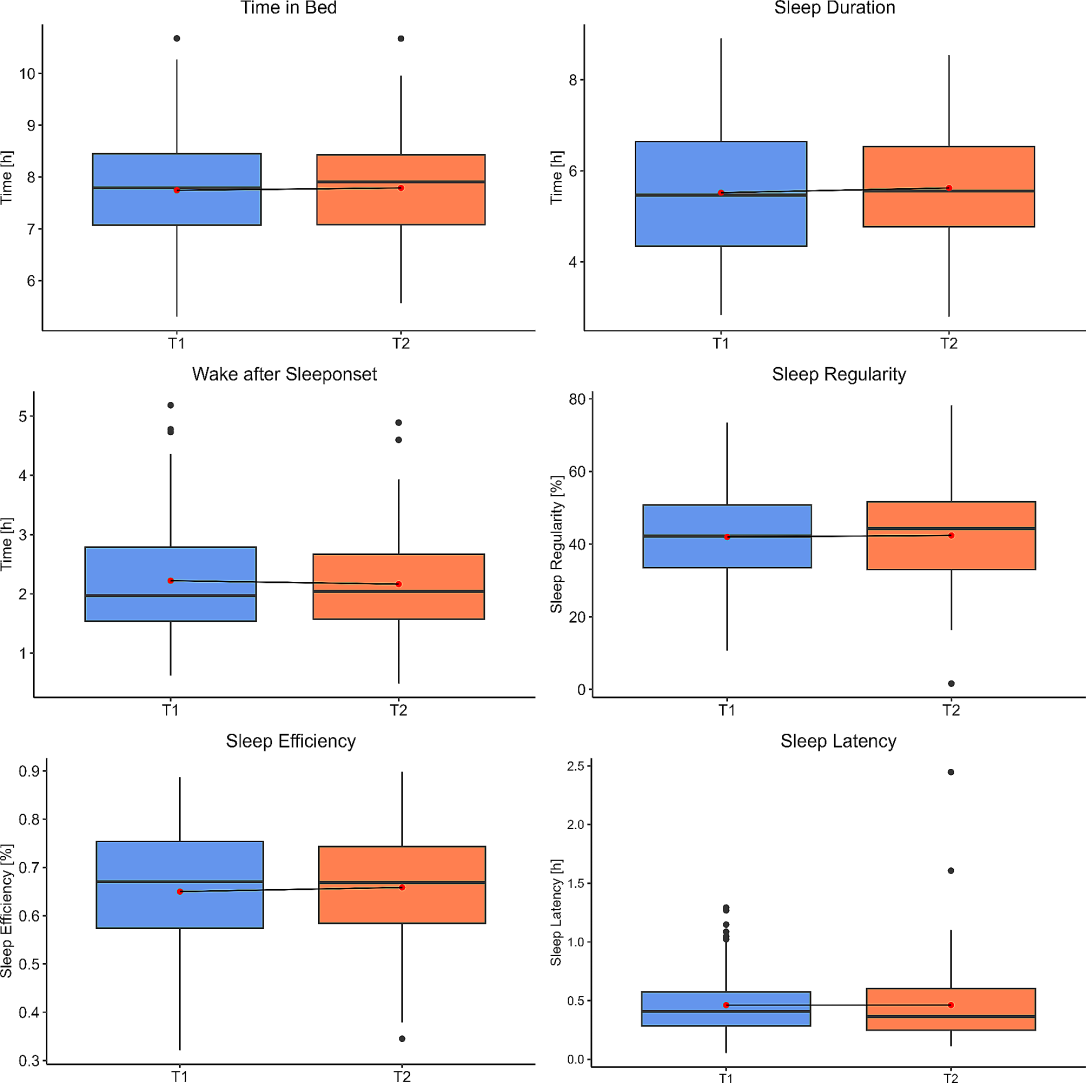
Distribution of the device assessed sleep parameters of post-COVID patients before (T1, blue) and after (T2, orange) inpatient rehabilitation. The median score is displayed as solid black line within the boxplot. The mean value is represented as red point

Table 3 and Fig. 4 are presenting the results of the measured sleep parameters before (T1) and after (T2) the rehabilitation. None of the reported parameters were changing significantly between the two timepoints. At T1, the patients were spending Mdn = 7.79 h (IQR: 7.08–8.46) in bed, the sleep duration had a median of 5.50 h (IQR: 4.42–6.65) and the patients were Mdn = 1.97 h (IQR: 1.50–2.77) awake during the night. Descriptively, two of these three parameters were marginally improving after rehabilitation with Mdn = 7.89 h (IQR: 7.05–8.44) in bed, and Mdn = 5.52 h (IQR: 4.84–6.40) sleeping. The WASO time was increasing in T2 with Mdn = 2.03 h (IQR: 1.55–2.68) being awake during the night. The sleep regularity has a median of 42.95% (IQR: 33.53–52.24) at T1 and Mdn = 41.17% (IQR: 31.95–51.06) at T2. The patients sleep efficiency is 67% (IQR: 58–76) in T1 and Mdn = 68% (IQR: 58–74) in T2. Before rehabilitation, the patients were falling asleep after Mdn = 0.4 h (IQR: 0.27–0.57). After rehabilitation, the sleep latency had a median of 0.36 h (IQR: 0.25–0.57). Within the current study sample, at T1, 37 patients were reaching a sleep duration of > 6 h per night, four patients had a WASO time shorter than 51 min, three patients were reaching a sleep efficiency higher or equal than 85% and 64 patients fell asleep within 30 min.

**Table 3:** Sleep parameters of post-COVID patients before (T1) and after (T2) inpatient rehabilitation program

	Timepoint		**z**	**p**	**r**
	T1	T2			
	Median (IQR)	Median (IQR)			
Time in Bed [h]	7.79 (7.08, 8.46)	7.89 (7.05, 8.44)	-0.601	0.923	-0.061
Sleep Duration [h]	5.50 (4.42, 6.65)	5.52 (4.84, 6.40)	-0.590	0.783	-0.060
WASO [h]	1.97 (1.50, 2.77)	2.03 (1.55, 2.68)	0.367	0.956	0.037
Sleep Regularity [%]	42.95 (33.53, 52.24)	41.17 (31.95, 51.06)	-0.204	0.507	-0.021
Sleep Efficiency [%]	67 (58, 76)	68 (58, 74)	-0.510	0.941	-0.052
Sleep Latency [h]	0.40 (0.27, 0.57)	0.36 (0.25, 0.57)	1.250	0.590	0.126

### Group differences

The paired study sample consist of 27 male and 71 female patients. The Wilcoxon rank sum test revealed a significant difference in inactivity time before rehabilitation between male (Mdn = 14.77 h IQR: 13.46–15.66) and female (Mdn = 13.70 h, IQR: 12.82–14.93, *p* = 0.038) patients (additional file 1, Table A1). After rehabilitation male patients reduced their inactivity time to Mdn = 14.43 h (IQR: 12.98–15.15), whereas female patients were staying almost at the same inactivity level (Mdn = 13.80 h, IQR: 12.60–15.30). The difference between these two groups was not significant at T2 (*p* > 0.05). Regarding the other PA levels, there are no further significant differences between male and female patients (*p* > 0.05). There are significant differences within the sleep parameters between male and female patients. At T1, female patients show a longer duration in bed during the night (Mdn = 7.85 h, IQR: 7.43–8.46) than male patients (Mdn = 7.12 h, IQR: 6.18–8.15, *p* = 0.012). Furthermore, female patients have a longer sleep duration at T1 (Mdn = 5.69 h, IQR: 4.86–6.65) and T2 (Mdn = 5.71 h, IQR: 5.03–6.66) than male patients (T1: Mdn = 4.51 h, IQR: 3.90–5.63, *p* = 0.014; T2: Mdn = 4.99 h, IQR: 4.26–6.03, *p* = 0.030). Female and male patients do not differ significantly in the variables WASO, sleep regularity, sleep efficiency and sleep latency (*p* > 0.05). For a detailed description of these variables see Table A2 in the additional file 1. No significant differences over time were observed for the groupwise comparison between male and female patients neither in the PA nor in the sleep parameters.

The analysis of the PA data resulted in a significant difference in the time spend in vigorous PA between younger (< 55 years, *n* = 46) and older (≥ 55 years, *n* = 52) patients (additional file 1, Table A3). Younger patients spend more time in vigorous PA during the day (Mdn = 0.15 min, IQR: 0.06–0.41) than older patients (Mdn = 0.07 min, IQR: 0.04 − 0.013, *p* = 0.010) before rehabilitation. Since younger patients decreased their vigorous PA time to Mdn = 0.13 min (IQR: 0.05–0.40) and older patients increased their vigorous PA time to Mdn = 0.08 min (IQR: 0.03–0.15), there wasn’t a significant group difference after rehabilitation (*p* > 0.05). Regarding age, no further group differences were identified in terms of PA. At T1, younger patients had a significantly longer sleep duration (Mdn = 5.91 h, IQR: 5.24–6.77) than older patients (Mdn = 5.91 h, IQR: 3.98–6.45, *p* = 0.012) (additional file 1, Table A4). After rehabilitation younger and older patients increased their sleep duration (younger patients: Mdn = 6.03 h, IQR: 5.04–6.78; older patients: Mdn = 5.23 h, IQR: 4.70–6.06). The difference in sleep duration was still significant between these age groups at T2 (*p* = 0.019). At T1, the measured WASO time was for younger patients Mdn = 1.89 h (1.52–2.52) and at T2 Mdn = 1.81 h (IQR: 1.36–2.37). For older patients the measured WASO Time was Mdn = 2.15 h (IQR: 1.64–2.96) at T1 and Mdn = 2.15 h (IQR: 1.62–2.85) at T2. The difference was significant at T2 (*p* = 0.014). Further, the results showed a significant group difference in sleep efficiency at both timepoints. At T1, younger patients had a higher sleep efficiency of Mdn = 69% (IQR: 64–76) than older patients with Mdn = 63% (IQR: 52–74, *p* = 0.021). At T2, the sleep efficiency of younger patients was Mdn = 70% (IQR: 64–78) and of older patients Mdn = 64% (IQR: 58–71, *p* = 0.013). When comparing the two age groups over time, no significant difference could be found.

Regarding the subgroups of the COVID-19 severity (mild-moderate: *n* = 69, severe-critical: *n* = 29), there is no significant difference neither in the PA data nor in any of the sleep parameters (additional file 1, Table A5 and Table A6). However, the data shows a slightly higher duration in inactivity time within the severe-critical COVID-19 group at T1 (Mdn = 14.73 h, IQR: 13.36–15.61) and T2 (Mdn = 13.93 h, IQR: 13.49–15.34) compared to the mild-moderate COVID-19 group (T1: Mdn = 13.70 h, IQR: 12.82–14.96, *p* > 0.05; T2: Mdn = 13.84 h, IQR: 12.50-15.02, *p* > 0.05). The sleep data seems to suggest, that mild COVID-19 patients have a longer sleep duration at both measurement points (T1: Mdn = 5.65 h, IQR: 4.61–6.65, T2: Mdn = 5.64 h, IQR: 4.90–6.60) compared to the severe-critical COVID-19 patients (T1: Mdn = 5.11 h, IQR: 407–642, *p* > 0.05; T2: Mdn = 5.23 h, IQR: 4.30–6.16, *p* > 0.05).

Group differences according to pre-existing conditions (cardiovascular diseases, respiratory diseases, mental illness, and metabolic diseases) were also calculated. The results did just show a significant difference in vigorous PA (T1) for cardiovascular disease (No: Mdn = 0.15 min, IQR: 0.06–0.37; Yes: Mdn = 0.07 min, IQR: 0.03–0.16, *p* = 0.033, *r* = 0.215), in vigorous PA (T2) for metabolic disease (No: Mdn = 0.13 min, IQR: 0.08–0.42; Yes: Mdn = 0.07 min, IQR: 0.03–0.18, *p* = 0.022, *r* = 0.232), and in light PA (T2) for respiratory disease (No: Mdn = 43.85 min, IQR: 31.13–55.89; Yes: Mdn = 56.56 min, IQR: 40.25–95.83, *p* = 0.020, *r*=-0.235). No group differences could be detected regarding the assessed sleep parameters for any pre-existing disease. For a detailed description see additional file 1, Table A7-A14.

### Interaction effects

The two-way ANOVA revealed the following significant interaction effects (Table A15-A22). A significant interaction effect was found between respiratory and mental pre-existing conditions regarding light PA at T1 (F (1) = 4.847, *p* = 0.030). Additionally, for moderate PA at T1, there was a significant interaction effect observed between cardiovascular pre-existing conditions and sex (F  (1)= 4.087, *p* = 0.046), as well as between metabolic pre-existing conditions and sex (F (1) = 7.725, *p* = 0.007). Furthermore, in the analysis of vigorous PA at T2, significant interaction effects were found regarding metabolic pre-existing conditions and sex (F (1) = 4.314, *p* = 0.041), as well as regarding metabolic and respiratory pre-existing conditions (F (1) = 5.666, *p* = 0.019).

In the analysis of sleep duration at T1, a significant interaction effect was found between sex and age (F (1) = 4.408, *p* = 0.038). Additionally, for sleep duration at T2, there was a significant interaction effect observed between the comorbidity metabolic disorder and acute COVID-19 severity (F (1) = 4.975, *p* = 0.028). Moreover, in the analysis of sleep regularity at T1, a significant interaction effect was detected between the comorbidities metabolic disorder and cardiovascular disease (F (1) = 4.284, *p* = 0.041). Finally, for sleep regularity at T2, a significant interaction effect was observed between sex and the comorbidity cardiovascular disease (F (1) = 7.100, *p* = 0.009).

## Discussion

The aim of the study was to examine the PA and sleep quality of patients with post-COVID before and after an inpatient post-COVID rehabilitation program. This study investigated the interested parameters by accelerometery as an objective and more valid measurement before and after rehabilitation in a longitudinal design. In summary, the patients didn’t experience a better sleep quality or being more physically active after rehabilitation. According to the groupwise comparison, some differences could be detected.

### Physical activity

The current data shows no significant difference in the PA behaviour of post-COVID patients before and after rehabilitation. At both measurement points, the patients are almost 14 h inactive during the day. The high inactivity time of the examined post-COVID patients can be explained by the high rates of fatigue and exercise intolerance at T1 and T2. At T1, 97% of patients are reporting symptoms of exercise intolerance and 91% symptoms of fatigue. This prevalence is not significantly decreasing after rehabilitation discharge. Further, sleep periods during the daytime are also classified as inactivity time and they are common in patients experiencing symptoms of fatigue. The accelerometer wearing position can also lead to higher inactivity time. If the patient is doing standing activities, where mainly the upper body and arms are moving, it is not possible for the accelerometer to detect this movement [75]. Compared with other studies including post-COVID patients, the assessed inactivity time is higher. Plekhanova, et al. [30] and Benitez, et al. [28] reported inactivity times up to 12.6 h per day. In contrast, in accelerometer studies with healthy study populations, the inactivity time is substantially lower than that of post-COVID patients with around 8.2 h per day [85, 86]. Even patients with COPD are spending less time inactive during the day (∼ 8 h) than the included post-COVID patients [87]. Previous research demonstrated that patients with chronic diseases have also a substantial lower amount of MVPA per day compared to healthy controls [88]. Contrary to the high inactivity time within the current study sample most of the patients could achieve the WHO recommendations for MVPA for adults with chronic diseases, which includes any bodily movement produced by skeletal muscles requiring an energy expenditure ≥ 3 METs [29]. Only six patients didn’t spend 150 min of MVPA during their week. This is in line with Benitez, et al. [28] but contrary to the results of Plekhanova, et al. [30] and van Bakel, et al. [31] which revealed, that the level of MVPA of post-COVID patients is not reaching the WHO recommendations. Considering the diverse and complex symptoms of post-COVID patients and the heterogeneity of the study results, nevertheless, it should be critically questioned whether the general WHO’s activity recommendations are suitable for such a specific sample, rather personalised concepts are needed. Furthermore, the WHO PA recommendations are mostly based on self-report data and a comparison with accelerometery data is difficult. The differences in the assessed PA may be explained by different accelerometer devices and methodological aspects. The choice of accelerometer wearing position, acceleration metrics and scoring algorithm influencing the PA estimates. Another factor could be the characteristic of the included study population. The current study has an interval between the acute SARS-CoV-2 infection and T1 of ∼ 402 days. Compared to the other studies, which examined the patients after three to six months after acute infection, the patients are suffering from the post-COVID symptomatic almost three-fold longer.

The current data suggests that the rehabilitation program should more address behavioural changes according to PA and activity-related health competencies. There is evidence, that a higher amount of PA could reduce the post-COVID symptomatic and support the recovery process [30]. More important, high inactivity times are associated with pro-inflammatory processes and have negative effects on functional capacity [34, 89]. High levels of MVPA may reduce the negative effects of sustained inactivity but the current evidence shows that it does not eliminate it completely [90, 91]. It is also necessary to change behaviour by interrupting long inactivity times with short periods of any PA intensity (e.g., walking during a phone call, standing) [92, 93]. According to current health psychology theories and models (e.g., health action process approach (HAPA) [94], transtheoretical model of behavioural change [95], The Physical Activity-related Health Competence [96]), the improvement of physical resilience and functional capacity during rehabilitation is necessary but not sufficient to induce behavioural change (increase in habitual PA and decrease in inactivity time) within the scope of aftercare. The Physical Activity-related Health Competence is an integrative model describing personal determinants of PA [96]. Apart from physical functionality, self-efficacy, knowledge, self-regulatory skills and exercise related attitudes are necessary to obtain health-related PA after rehabilitation in a long-term. To assess and strengthen the individual determinants of the Physical Activity-related Health Competence within clinical settings (e.g., during rehabilitation) may improve the rehabilitation outcomes in a long-term [97]. Thus, interventions during rehabilitation as well as in the aftercare process should include (app-based) monitoring of PA, strengthening exercises and endurance training and educational units about the effects of PA and inactivity on post-COVID patients’ health. Further, the patients should gain competencies about adapting the PA intensity to their current physical and mental state, maintaining or increasing PA after rehabilitation (in relation to HAPA [94]) and how to deal with internal and external barriers during the implementation of PA.

According to the groupwise comparison, male as well as older post-COVID patients tend to have a less physical active lifestyle than female and younger post-COVID patients. These results underline the findings of Plekhanova, et al. [30]. Particularly for older post-COVID patients, the promotion of a physically active lifestyle is important, as in addition to post-COVID disease, structural and physical barriers towards PA also increase with age [98].

In addition, the data suggests, that pre-existing comorbidities also influence the PA behaviour of the study population. This is in line with previous research in COPD patients. Sievi, et al. [99] and Mantoani, et al. [100] could show, that COPD patients with comorbidities have a lower level of PA than patients without any comorbidity. Thus, within the rehabilitation process, the level of PA of post-COVID patients with pre-existing conditions should be addressed, considering their existing resources and impairments. General, the significant interaction effects reveal complex relationships between pre-existing health conditions and demographic factors in influencing PA. Respiratory and mental conditions interact to affect light PA at T1, while cardiovascular and metabolic conditions interact with sex for moderate PA at T1. Similarly, metabolic conditions interact with sex and respiratory conditions for vigorous PA at T2. These findings highlight the importance of personalized rehabilitation approaches considering both health status and demographic factors to promote PA in post-COVID patients. However, the variability within the compared groups was high and the sample size was not well balanced. Therefore, more longitudinal data with longer follow-up intervals and focus on the intraindividual (within-subject) analysis is needed in the future.

### Sleep quality

Like PA no changes in sleep quality of post-COVID patients could be detected before and after rehabilitation. Overall, the patients show poor sleep quality. Several reported parameters are below the recommendations for adults of the National Sleep Foundation [83]. A good sleep quality is characterized by a sleep duration of six to 10 h, a sleep latency of less than 30 min, a sleep efficiency over 85%, and a WASO-time less than 51 min. At T1 as well as at T2 the patients have a median sleep duration of 5.5 h, the sleep efficiency is Mdn = 67% and the median of the WASO time is ∼ 2 h. Only the sleep latency is within the recommended range with a median of 24 min. This is in line with the subjective assessed sleep quality. At T1 79% of patients indicate to suffer from sleep disturbances. This prevalence is not significantly decreasing after rehabilitation discharge. The unchanged sleep quality after rehabilitation may be explained by the measurement procedure. The patients wore the accelerometer and filled out the questionnaires two weeks after rehabilitation discharge. Possibly, the patients first had to get used to their home environment and everyday life again after being discharged from rehabilitation. Some persons were still on sick leave a few days after rehabilitation. Thus, the time of measurement could have fallen on the first working days, which may also result in a change in the sleep-wake rhythm. Another explanation for the ongoing poor sleep quality of post-COVID patients is the persistently high burden of disease after rehabilitation. The prevalences of e.g., exercise intolerance, fatigue, joint and muscle pain, and mental disorders are high with 60–87% of patients still suffering from these symptoms. Previous research reported a poor sleep quality of post-COVID patients, too [53, 101, 102]. Jarosch, et al. [53] examined sleep quality of post-COVID patients with polysomnography and compared the results with healthy controls. The sleep quality of post-COVID patients was significantly impaired. Furthermore, the post-COVID patients in the study by Mekhael, et al. [103] experienced a significantly shorter sleep duration compared to healthy controls. In general, regarding the impairment of the immune system functionality due to sleep deprivation and poor sleep quality [54, 56, 104], the post-COVID rehabilitation program should focus more on a better treatment of sleep disturbances. Potential approaches to address sleep disturbances during rehabilitation is sleep-related psychoeducation, cognitive behaviour therapy, inducing sleep structuring techniques, and mindfulness-based interventions [81, 105]. It is also known that self-observation of the own sleep induces positive effects on sleep quality [106]. Within the rehabilitation process, it is important to identify and consequently treat the main symptoms behind the sleep disorders. They are often linked to symptoms of depression or anxiety, requiring long-term psychological support to permanently counteract the sleep disorders. Furthermore, for post-COVID patients with ME/CFS, the PACING technique may also lead to an improvement in sleep quality [5, 16, 107].

The data shows sex related group differences in sleep parameters. Even if both groups were showing a poor sleep quality, female patients did spend significantly more time in bed during the night and slept longer at both measurement points. In general, females are more likely to need more sleep during the night caused by a different hormonal state [82, 108]. However, the difference could be explained by higher prevalence of mental impairments and fatigue in women in the current sample. The analysis of Müller, et al. [38] with the same cohort revealed sex-based differences in the fatigue symptomatic, which may also lead to longer sleep duration and time in bed. Nevertheless, neither male nor female patients show sleep parameters indicating a good sleep quality. Regarding age, younger patients (< 55 years) have a significant longer sleep duration with around 6 h per night at T1 and T2. Patients ≥ 55 years have a sleep duration of around 5 h per night at T1 and T2. Further, younger patients have a significantly better sleep efficiency (∼ 70%) than older patients (∼ 64%) before and after rehabilitation. According to the literature, the sleep duration is decreasing with increasing age and the deep sleep stage gets shorter [109]. Older patients wake up more often during the night and the current data seems to confirm this observation as the difference in WASO time is significantly shorter in younger patients than in older. The analysis of the interaction effects illustrates the need to consider pre-existing conditions when treating sleep disorders, as these interact with demographic factors, among others. It is important to recognise that the general findings from the literature may be based on broad populations studies. However, our study focuses on a specific population and a limited time period. For more profound insights, further longitudinal studies may be necessary to understand the specific mechanisms and factors influencing sleep quality in post-COVID patients.

### Strength and limitations

There are several strengths and limitations within the study. The device-based assessment of PA and sleep quality strengthen the validity of the given results. Thus, through the results it is possible to gain more knowledge about the PA behaviour and sleep quality of post-COVID patients undergoing a rehabilitation program.

It is important to note, that this observational cohort study did not include any control group, which should be considered when interpreting the results. Further investigations should include a control group with healthy controls, patients with other chronic diseases or post-COVID patients without rehabilitation intervention to strengthen the validity of the current results.

The selective characteristics of the examined sample is also limiting the generalizability of the results to a broader post-COVID population. First, most of the patients were working within the healthcare sector. Due to rotating working hours and nights shifts this is influencing sleep quality [55]. In further investigations the assessment of preexisting sleep problems is necessary. Second, the patients were suffering from post-COVID a long time (∼ 400 days). Hayden, et al. [110] stated, that rehabilitation program is more effective soon after acute COVID-19. Also, there may be a selection bias within our study, since severely affected post-COVID patients may have had more intrinsic motivation to participate and share their data, potentially skewing the results.

In general, further studies should focus more on external factors such as lifestyle, working and environmental conditions and psychological stress, as they are likely to influence both PA behaviour and sleep quality.

The available data provides a very general overview of the PA behaviour of post-COVID patients and does not depict specific forms, patterns, and intensities of PA. This emphasises the need for more precise data collection to enable a more differentiated analysis of PA behaviour of post-COVID patients. The sensor wearing position on the right waist is also limiting the results since movements with the upper extremities could not be detected and the distinguishing between different body positions (e.g., sitting vs. standing) is not possible. Also, the time interval between the two measurement points is quite short, as effects on PA and sleep quality may only become apparent in the longer term, and thus, another measurement point e.g., 12 months after rehabilitation is needed. Additionally, this allows longitudinal analyses over three measurement points and to estimate e.g., within-person impacts of the rehabilitation phase and post-rehabilitation phase on PA and sleep quality.

At last, the rehabilitation program was not explicitly designed to improve patients’ sleep quality. However, since it follows a holistic approach and sleep disorders are one of the most common post-COVID symptoms, a separate examination of patients’ sleep is legitimate. The results encourage to implement interventions such as sleep-related psychoeducation and cognitive behaviour therapy in future rehabilitation and aftercare-process of post-COVID patients.

### Conclusions

Despite the significant decrease in the prevalence of post-COVID symptoms after rehabilitation, no improvement in objectively assessed PA behaviour and sleep quality could be detected after inpatient post-COVID rehabilitation. The patients are highly inactive with around 14 h per day. However, most patients achieve more than 150 min per week MVPA, possibly compensating for some of the negative health outcomes of too much inactivity time. Compared to sleep recommendations, the post-COVID patients have a bad sleep quality before and after rehabilitation. This is in line with the self-reported prevalence of sleep disturbances. Overall, the change of PA behaviour and how to acquire a good sleep hygiene should be addressed during inpatient rehabilitation in a more targeted and well-founded manner. The acquisition of competences to maintain a physical active lifestyle through educational units and active training should be focussed within rehabilitation in relation to current health psychological models and theories. Within the aftercare process (app-based) monitoring and promotion of PA could help, to maintain an active lifestyle as well. In addition, the rehabilitation program should address the high prevalence of sleep disorders within post-COVID patients by focusing more on established techniques such as sleep-related psychoeducation, cognitive behavioural therapy, and sleep structuring techniques. Psychological support for the therapy of potential underlying symptoms is also advisable. Future research should examine the effectiveness of such interventions and observe the PA behaviour and sleep quality of post-COVID patients in a longitudinal and controlled design. Furthermore, qualitative research seems beneficial to analyse and reconstruct the recovery process of a person, attempts to reveal individual paths, trajectories, and coping strategies and at the same time to identify factors why adopting a more active lifestyle and recovery (e.g. better sleep quality) was successful or failed so far.

### Electronic supplementary material

Below is the link to the electronic supplementary material.

Supplementary Material 1

## Data Availability

The data are available from the corresponding author upon request.

## Abbreviations

COVID-19: Coronavirus disease 2019
DRKS: German Register for Clinical Studies
ENMO: Euclidean Norm Minus One with negative values rounded to zero
ENT: Ear-nose-throat
ICU: Intensive care unit
HAPA: Health Action Process Approach
M: Mean
Mdn: Median
ME/CFS: Myalgic encephalomyelitis/chronic fatigue syndrome
MET: Metabolic equivalent of task
mg: Milligravitational
PA: Physical Activity
SARS-CoV-2: Severe acute respiratory syndrome coronavirus type 2
WASO: Wake after sleep onset
WHO: World health organization
