# Supplementary material for: Device-assessed physical activity and sleep quality of post-COVID patients undergoing a rehabilitation program
Source: BMC Sports Sci Med Rehabil. 2024 May 29;16:122. doi: 10.1186/s13102-024-00909-2 (PMC11134673; doi:10.1186/s13102-024-00909-2)
Supplement: Supplementary file 1 — Supplementary Material 1 [file 13102_2024_909_MOESM1_ESM.docx]

Additional file 1

| Table A1: Groupwise comparison of PA of male and female post-COVID patients at timepoint T1 and T2. | | | | | |
| --- | --- | --- | --- | --- | --- |
|  | **Sex** | |  |  |  |
|  | male  (n=27) | female  (n=71) |  |  |  |
|  | Median (IQR) | Median (IQR) | **z** | **p** | **r** |
| Inactive [h] T1 | 14.77  (13.46, 15.66) | 13.70  (12.82, 14.93) | 2.080 | 0.038 | 0.210 |
| Inactive [h] T2 | 14.43  (12.98, 15.15) | 13.80  (12.60, 15.03) | 0.874 | 0.386 | 0.088 |
| Light Activity [min] T1 | 41.49  (34.48, 78.51) | 48.77  (36.31, 72.99) | -0.548 | 0.562 | -0.055 |
| Light Activity [min] T2 | 40.25  (33.06, 57.11) | 48.70  (34.28, 78.29) | -1.420 | 0.157 | -0.143 |
| Moderate Activity [min] T1 | 60.01  (34.78, 79.89) | 48.59  (38.60, 68.43) | 0.632 | 0.530 | 0.064 |
| Moderate Activity [min] T2 | 49.52  (35.28, 80.56) | 52.62  (38.24, 72.52) | -0.155 | 0.880 | -0.016 |
| Vigorous Activity [min] T1 | 0.08  (0.04, 0.15) | 0.11  (0.04, 0.31) | -0.768 | 0.445 | -0.078 |
| Vigorous Activity [min] T2 | 0.08  (0.05, 0.18) | 0.10  (0.04, 0.35) | -0.334 | 0.741 | -0.034 |

| Table A2: Groupwise comparison of sleep parameters of male and female post-COVID patients at timepoint T1 and T2. | | | | | |
| --- | --- | --- | --- | --- | --- |
|  | **Sex** | |  |  |  |
|  | male  (n=27) | female  (n=71) |  |  |  |
|  | Median (IQR) | Median (IQR) | **z** | **p** | **r** |
| Time in Bed [h] T1 | 7.12  (6.18, 8.15) | 7.85  (7.43, 8.46) | -2.510 | 0.012 | -0.254 |
| Time in Bed [h] T2 | 7.56  (6.23, 8.25) | 8.01  (7.21, 8.47) | -1.860 | 0.063 | -0.188 |
| Sleep duration [h] T1 | 4.51  (3.90, 5.63) | 5.69  (4.86, 6.65) | -2.470 | 0.014 | -0.250 |
| Sleep duration [h] T2 | 4.99  (4.26, 6.03) | 5.71  (5.03, 6.66) | -2.170 | 0.030 | -0.219 |
| WASO [h] T1 | 2.20  (1.65, 2.93) | 1.96  (1.51, 2.75) | 0.763 | 0.448 | 0.077 |
| WASO [h] T2 | 2.02  (1.59, 2.73) | 2.06  (1.57, 2.53) | 0.159 | 0.877 | 0.016 |
| Sleep Regularity [%] T1 | 41.46  (33.78, 47.70) | 42.50  (33.36, 51.90) | -0.656 | 0.514 | -0.066 |
| Sleep Regularity [%] T2 | 41.17  (30.03, 51.31) | 44.40  (34.36, 51.84) | -0.807 | 0.422 | -0.082 |
| Sleep Efficiency [%] T1 | 0.61  (0.54, 0.71) | 0.69  (0.60, 0.76) | -1.860 | 0.063 | -0.188 |
| Sleep Efficiency [%] T2 | 0.63  (0.56, 0.73) | 0.68  (0.59, 0.76) | -1.320 | 0.189 | -0.133 |
| Sleep Latency [h] T1 | 0.37  (0.28, 0.57) | 0.41  (0.29, 0.57) | -0.306 | 0.763 | -0.031 |
| Sleep Latency [h] T2 | 0.33  (0.24, 0.65) | 0.38  (0.25, 0.58) | -0.406 | 0.688 | -0.041 |

| Table A3: Groupwise comparison of PA of younger and older post-COVID patients at timepoint T1 and T2. | | | | | |
| --- | --- | --- | --- | --- | --- |
|  | **Age** | |  |  |  |
|  | younger than  55 years  (n=46) | at least 55 years  (n=52) |  |  |  |
|  | Median (IQR) | Median (IQR) | **z** | **p** | **r** |
| Inactive [h] T1 | 13.55  (12.68, 14.89) | 14.01  (13.10, 15.43) | -1.840 | 0.067 | -0.186 |
| Inactive [h] T2 | 13.79  (12.49, 15.01) | 13.92  (13.00, 15.08) | -0.840 | 0.403 | -0.085 |
| Light Activity [min] T1 | 48.87  (38.02, 73.36) | 41.04  (33.12, 74.26) | 1.050 | 0.294 | 0.106 |
| Light Activity [min] T2 | 49.82  (33.03, 91.34) | 46.83  (33.71, 60.53) | 0.733 | 0.466 | 0.074 |
| Moderate Activity [min] T1 | 54.40  (40.38, 69.97) | 47.52  (34.59, 71.74) | 0.669 | 0.506 | 0.068 |
| Moderate Activity [min] T2 | 55.24  (36.18, 72.62) | 48.22  (35.95, 73.38) | 0.712 | 0.479 | 0.072 |
| Vigorous Activity [min] T1 | 0.15  (0.06, 0.41) | 0.07  (0.04, 0.13) | 2.600 | 0.010 | 0.263 |
| Vigorous Activity [min] T2 | 0.13  (0.05, 0.40) | 0.08  (0.03, 0.15) | 1.620 | 0.107 | 0.164 |

| Table A4: Groupwise comparison of sleep parameters of younger and older post-COVID patients at timepoint T1 and T2. | | | | | |
| --- | --- | --- | --- | --- | --- |
|  | **Age** | |  |  |  |
|  | younger than 55 years  (n=44) | at least 55 years  (n=54) |  |  |  |
|  | Median (IQR) | Median (IQR) | **z** | **p** | **r** |
| Time in Bed [h] T1 | 7.85  (7.32, 8.71) | 7.76  (6.95, 8.24) | 1.120 | 0.262 | 0.113 |
| Time in Bed [h] T2 | 7.97  (7.04, 8.36) | 7.80  (7.09, 8.46) | 0.371 | 0.713 | 0.037 |
| Sleep duration [h] T1 | 5.91  (5.24, 6.77) | 5.00  (3.98, 6.45) | 2.510 | 0.012 | 0.254 |
| Sleep duration [h] T2 | 6.03  (5.04, 6.78) | 5.23  (4.70, 6.06) | 2.350 | 0.019 | 0.237 |
| WASO [h] T1 | 1.89  (1.52, 2.52) | 2.22  (1.64, 3.10) | -1.780 | 0.076 | -0.180 |
| WASO [h] T2 | 1.81  (1.36, 2.37) | 2.21  (1.74, 2.85) | -2.460 | 0.014 | -0.248 |
| Sleep Regularity [%] T1 | 40.96  (31.65, 51.10) | 43.68  (33.74, 49.97) | -0.600 | 0.551 | -0.061 |
| Sleep Regularity [%] T2 | 41.11  (31.26, 51.89) | 44.85  (34.37, 50.86) | -0.164 | 0.872 | -0.017 |
| Sleep Efficiency [%] T1 | 0.69  (0.64, 0.76) | 0.63  (0.52, 0.74) | 2.300 | 0.021 | 0.232 |
| Sleep Efficiency [%] T2 | 0.70  (0.64, 0.78) | 0.64  (0.58, 0.71) | 2.500 | 0.013 | 0.253 |
| Sleep Latency [h] T1 | 0.43  (0.32, 0.53) | 0.37  (0.27, 0.64) | -0.089 | 0.932 | -0.009 |
| Sleep Latency [h] T2 | 0.30  (0.23, 0.48) | 0.41  (0.29, 0.64) | -1.900 | 0.058 | -0.192 |

| Table A5: Groupwise comparison of PA of patients with a mild-moderate COVID-19 and a severe-critical COVID-19 at timepoint T1 and T2. | | | | | |
| --- | --- | --- | --- | --- | --- |
|  | **COVID-19 Severity** | |  |  |  |
|  | mild-moderate  (n=71) | severe-critical (n=27) |  |  |  |
|  | Median (IQR) | Median (IQR) | **z** | **p** | **r** |
| Inactive [h] T1 | 13.70  (12.82, 14.96) | 14.73  (13.36, 15.61) | 0.180 | 0.105 | 0.018 |
| Inactive [h] T2 | 13.84  (12.50, 15.02) | 13.93  (13.49, 15.34) | -1.310 | 0.192 | -0.132 |
| Light Activity [min] T1 | 48.41  (34.48, 73.32) | 47.93  (36.88, 73.98) | -0.243 | 0.811 | -0.025 |
| Light Activity [min] T2 | 46.74  (33.75, 71.22) | 50.85  (32.30, 63.93) | 0.211 | 0.836 | 0.021 |
| Moderate Activity [min] T1 | 51.63  (36.07, 69.77) | 63.25  (39.57, 74.86) | -0.775 | 0.441 | -0.078 |
| Moderate Activity [min] T2 | 49.73  (35.99, 71.24) | 56.74  (37.06, 77.16) | -0.600 | 0.551 | -0.061 |
| Vigorous Activity [min] T1 | 0.12  (0.05, 0.31) | 0.08  (0.03, 0.20) | 1.070 | 0.286 | 0.108 |
| Vigorous Activity [min] T2 | 0.12  (0.04, 0.32) | 0.07  (0.02, 0.21) | 1.230 | 0.221 | 0.124 |

| Table A6: Groupwise comparison of sleep parameters of patients with a mild-moderate COVID-19 and a severe-critical COVID-19 at timepoint T1 and T2. | | | | | |
| --- | --- | --- | --- | --- | --- |
|  | **COVID-19 Severity** | |  |  |  |
|  | mild-moderate (n=69) | severe-critical (n=29) |  |  |  |
|  | Median (IQR) | Median (IQR) | **z** | **p** | **r** |
| Time in Bed [h] T1 | 7.79  (7.12, 8.51) | 7.83  (6.32, 8.19) | 1.130 | 0.259 | 0.114 |
| Time in Bed [h] T2 | 7.98  (7.27, 8.48) | 7.46  (6.88, 8.19) | 1.660 | 0.097 | 0.168 |
| Sleep duration [h] T1 | 5.65  (4.51, 6.65) | 5.11  (4.07, 6.42) | 1.180 | 0.241 | 0.119 |
| Sleep duration [h] T2 | 5.64  (4.90, 6.60) | 5.23  (4.30, 6.16) | 1.600 | 0.111 | 0.162 |
| WASO [h] T1 | 2.10  (1.53, 2.80) | 1.87  (1.57, 2.57) | 0.241 | 0.812 | 0.024 |
| WASO [h] T2 | 2.02  (1.55, 2.63) | 2.11  (1.63, 2.78) | -0.689 | 0.493 | -0.070 |
| Sleep Regularity [%] T1 | 40.47  (33.08, 49.78) | 46.35  (39.20, 54.32) | -1.890 | 0.060 | -0.191 |
| Sleep Regularity [%] T2 | 40.82  (30.25, 51.69) | 44.89  (37.16, 51.06) | -1.350 | 0.178 | -0.136 |
| Sleep Efficiency [%] T1 | 0.67  (0.58, 0.76) | 0.67  (0.53, 0.73) | 0.673 | 0.503 | 0.068 |
| Sleep Efficiency [%] T2 | 0.68  (0.59, 0.75) | 0.64  (0.58, 0.72) | 0.965 | 0.336 | 0.097 |
| Sleep Latency [h] T1 | 0.40  (0.27, 0.55) | 0.49  (0.30, 0.64) | -1.120 | 0.266 | -0.113 |
| Sleep Latency [h] T2 | 0.33  (0.24, 0.56) | 0.41  (0.33, 0.61) | -1.260 | 0.209 | -0.127 |

| Table A7: Groupwise comparison of PA of patients with pre-existing cardiovascular disease and without a pre-existing cardiovascular disease at timepoint T1 and T2. | | | | | |
| --- | --- | --- | --- | --- | --- |
|  | **Pre-existing cardiovascular disease** | |  |  |  |
|  | No  (n=47) | Yes  (n=51) |  |  |  |
|  | Mdn (IQR) | Mdn (IQR) | z | **p** | **r** |
| Inactive [h] T1 | 13.70  (12.82, 15.14) | 14.05  (13.17, 15.29) | -0.985 | 0.326 | -0.100 |
| Inactive [h] T2 | 13.97  (12.63, 15.03) | 13.80  (12.64, 15.11) | 0.060 | 0.955 | 0.006 |
| Light Activity [min] T1 | 47.94  (37.22, 73.98) | 47.93  (32.47, 73.32) | 0.594 | 0.555 | 0.060 |
| Light Activity [min] T2 | 48.76  (37.90, 66.79) | 43.85  (32.71, 71.64) | 0.480 | 0.634 | 0.048 |
| Moderate Activity [min] T1 | 53.70  (39.69, 71.57) | 51.63  (34.47, 71.03) | 1.010 | 0.313 | 0.102 |
| Moderate Activity [min] T2 | 53.65  (39.30, 75.60) | 49.52  (33.93, 68.80) | 1.300 | 0.196 | 0.131 |
| Vigorous Activity [min] T1 | 0.15  (0.06, 0.37) | 0.07  (0.03, 0.16) | 2.130 | 0.033 | 0.215 |
| Vigorous Activity [min] T2 | 0.11  (0.04, 0.39) | 0.08  (0.04, 0.19) | 0.630 | 0.531 | 0.064 |

| Table A8: Groupwise comparison of sleep parameters of patients with pre-existing cardiovascular disease and without pre-existing cardiovascular disease at timepoint T1 and T2. | | | | | |
| --- | --- | --- | --- | --- | --- |
|  | **Pre-existing cardiovascular disease** | |  |  |  |
|  | No  (n=46) | Yes  (n=52) |  |  |  |
|  | Mdn (IQR) | Mdn (IQR) | **z** | **p** | **r** |
| Time in Bed [h] T1 | 7.89  (7.41, 8.30) | 7.45  (6.83, 8.51) | 1.250 | 0.214 | 0.126 |
| Time in Bed [h] T2 | 7.92  (7.05, 8.32) | 7.85  (7.23, 8.45) | -0.409 | 0.685 | -0.041 |
| Sleepduration [h] T1 | 5.91  (4.54, 6.64) | 5.30  (4.16, 6.53) | 1.270 | 0.206 | 0.128 |
| Sleepduration [h] T2 | 5.56  (4.92, 6.60) | 5.54  (4.68, 6.39) | 0.545 | 0.588 | 0.055 |
| Wake after Sleeponset [h] T1 | 1.88  (1.51, 2.75) | 2.23  (1.64, 2.86) | -0.975 | 0.331 | -0.098 |
| Wake after Sleeponset [h] T2 | 1.94  (1.57, 2.36) | 2.11  (1.56, 2.78) | -0.851 | 0.397 | -0.086 |
| Sleep Regularity [%] T1 | 42.83  (33.19, 50.17) | 42.20  (33.90, 51.78) | -0.434 | 0.667 | -0.044 |
| Sleep Regularity [%] T2 | 44.38  (32.14, 52.03) | 42.58  (33.04, 50.18) | 0.612 | 0.543 | 0.062 |
| Sleep Efficiency [%] T1 | 0.70  (0.59, 0.76) | 0.65  (0.57, 0.72) | 1.340 | 0.182 | 0.135 |
| Sleep Efficiency [%] T2 | 0.69  (0.60, 0.75) | 0.65  (0.58, 0.73) | 0.762 | 0.448 | 0.077 |
| Sleep Latency [h] T1 | 0.38  (0.26, 0.57) | 0.42  (0.33, 0.60) | -0.673 | 0.503 | -0.068 |
| Sleep Latency [h] T2 | 0.36  (0.23, 0.56) | 0.37  (0.26, 0.63) | -0.886 | 0.377 | -0.082 |

| Table A9: Groupwise comparison of PA of patients with pre-existing respiratory disease and without pre-existing respiratory disease at timepoint T1 and T2. | | | | | |
| --- | --- | --- | --- | --- | --- |
|  | **Pre-existing respiratory disease** | |  |  |  |
|  | No  (n=57) | Yes  (n=41) |  |  |  |
|  | Mdn (IQR) | Mdn (IQR) | **z** | **p** | **r** |
| Inactive [h] T1 | 13.73  (12.96, 15.27) | 14.21  (12.91, 14.92) | -0.471 | 0.948 | -0.048 |
| Inactive [h] T2 | 13.85  (13.05, 15.15) | 13.80  (12.23, 14.99) | 1.130 | 0.261 | 0.114 |
| Light Activity [min] T1 | 48.41  (36.84, 64.44) | 43.38  (33.76, 82.63) | 0.090 | 0.931 | 0.009 |
| Light Activity [min] T2 | 43.85  (31.13, 55.89) | 56.56  (40.25, 95.83) | -2.330 | 0.020 | -0.235 |
| Moderate Activity [min] T1 | 51.63  (36.55, 70.20) | 53.03  (39.90, 74.44) | -0.681 | 0.498 | -0.069 |
| Moderate Activity [min] T2 | 48.39  (35.40, 69.67) | 55.14  (39.03, 73.62) | -0.911 | 0.364 | -0.092 |
| Vigorous Activity [min] T1 | 0.07  (0.03, 0.23) | 0.13  (0.06, 0.31) | -1.890 | 0.059 | -0.191 |
| Vigorous Activity [min] T2 | 0.10  (0.02, 0.27) | 0.10  (0.05, 0.38) | -0.598 | 0.552 | -0.060 |

| Table A10: Groupwise comparison of sleep parameters of patients with pre-existing respiratory disease and without pre-existing respiratory disease at timepoint T1 and T2. | | | | | |
| --- | --- | --- | --- | --- | --- |
|  | **Pre-existing respiratory disease** | |  |  |  |
|  | No  (n=57) | Yes  (n=41) |  |  |  |
|  | Mdn (IQR) | Mdn (IQR) | **z** | **p** | **r** |
| Time in Bed [h] T1 | 7.85  (7.12, 8.75) | 7.74  (7.02, 8.19) | 1.380 | 0.170 | 0.139 |
| Time in Bed [h] T2 | 7.98  (7.12, 8.46) | 7.88  (7.01, 8.29) | 0.634 | 0.529 | 0.064 |
| Sleepduration [h] T1 | 5.86  (4.73, 6.81) | 5.30  (4.14, 6.30) | 1.810 | 0.070 | 0.183 |
| Sleepduration [h] T2 | 5.62  (4.75, 6.72) | 5.52  (4.99, 6.16) | 0.274 | 0.787 | 0.028 |
| Wake after Sleeponset [h] T1 | 1.96  (1.71, 2.69) | 2.20  (1.46, 3.03) | -0.699 | 0.487 | -0.071 |
| Wake after Sleeponset [h] T2 | 2.11  (1.59, 2.78) | 2.00  (1.55, 2.63) | 0.663 | 0.510 | 0.067 |
| Sleep Regularity [%] T1 | 42.07  (34.03, 50.18) | 44.40  (33.04, 51.56) | -0.335 | 0.740 | -0.034 |
| Sleep Regularity [%] T2 | 44.34  (31.52, 51.69) | 43.99  (34.56, 51.06) | -0.241 | 0.812 | -0.024 |
| Sleep Efficiency [%] T1 | 0.68  (0.60, 0.76) | 0.64  (0.55, 0.74) | 1.070 | 0.286 | 0.108 |
| Sleep Efficiency [%] T2 | 0.68  (0.58, 0.76) | 0.66  (0.59, 0.73) | 0.155 | 0.880 | 0.016 |
| Sleep Latency [h] T1 | 0.41  (0.29, 0.59) | 0.41  (0.29, 0.56) | 0.342 | 0.735 | 0.035 |
| Sleep Latency [h] T2 | 0.38  (0.24, 0.61) | 0.36  (0.26, 0.57) | -0.068 | 0.948 | -0.006 |

| Table A11: Groupwise comparison of PA of patients with pre-existing mental illness and without pre-existing mental illness at timepoint T1 and T2. | | | | | |
| --- | --- | --- | --- | --- | --- |
|  | **Pre-existing mental illness** | |  |  |  |
|  | No  (n=79) | Yes  (n=19) |  |  |  |
|  | Mdn (IQR) | Mdn (IQR) | **z** | **p** | **r** |
| Inactive [h] T1 | 13.82  (12.89, 15.18) | 14.34  (13.09, 15.15) | -0.499 | 0.621 | -0.050 |
| Inactive [h] T2 | 13.84  (12.66, 15.02) | 14.22  (12.55, 15.29) | -0.373 | 0.713 | -0.038 |
| Light Activity [min] T1 | 48.66  (36.78, 73.98) | 40.58  (30.21, 67.96) | 1.160 | 0.246 | 0.117 |
| Light Activity [min] T2 | 48.06  (34.28, 71.03) | 44.71  (33.16, 63.93) | 0.364 | 0.719 | 0.037 |
| Moderate Activity [min] T1 | 54.33  (39.22, 72.56) | 39.90  (34.48, 64.70) | 1.510 | 0.133 | 0.153 |
| Moderate Activity [min] T2 | 53.32  (38.57, 74.69) | 46.20  (30.34, 60.09) | 1.660 | 0.098 | 0.168 |
| Vigorous Activity [min] T1 | 0.11  (0.05, 0.29) | 0.08  (0.02, 0.17) | 1.320 | 0.188 | 0.133 |
| Vigorous Activity [min] T2 | 0.08  (0.04, 0.38) | 0.11  (0.04, 0.18) | 0.476 | 0.637 | 0.048 |

| Table A12: Groupwise comparison of sleep parameters of patients with pre-existing mental illness and without pre-existing mental illness at timepoint T1 and T2. | | | | | |
| --- | --- | --- | --- | --- | --- |
|  | **Pre-existing mental illness** | |  |  |  |
|  | No  (n=80) | Yes  (n=18) |  |  |  |
|  | Mdn (IQR) | Mdn (IQR) | **z** | **p** | **r** |
| Time in Bed [h] T1 | 7.74  (7.03, 8.35) | 8.03  (7.44, 8.49) | -0,963 | 0.338 | -0,097 |
| Time in Bed [h] T2 | 7.80  (7.06, 8.45) | 8.20  (7.31, 8.38) | -0,922 | 0.359 | -0,093 |
| Sleepduration [h] T1 | 5.55  (4.39, 6.65) | 5.30  (4.43, 6.40) | 0,417 | 0.680 | 0,042 |
| Sleepduration [h] T2 | 5.56  (4.87, 6.59) | 5.57  (4.68, 6.34) | 0,050 | 0.963 | 0,005 |
| Wake after Sleeponset [h] T1 | 1.99  (1.49, 2.75) | 1.96  (1.72, 3.38) | -0,973 | 0.333 | -0,098 |
| Wake after Sleeponset [h] T2 | 2.02  (1.57, 2.63) | 2.19  (1.68, 2.85) | -0,771 | 0.444 | -0,078 |
| Sleep Regularity [%] T1 | 42.38  (33.90, 51.27) | 41.31  (33.19, 49.37) | 0,450 | 0.656 | 0,045 |
| Sleep Regularity [%] T2 | 44.30  (33.04, 51.89) | 44.19  (29.38, 50.20) | 0,367 | 0.717 | 0,037 |
| Sleep Efficiency [%] T1 | 0.67  (0.59, 0.75) | 0.65  (0.52, 0.76) | 0,784 | 0.435 | 0,079 |
| Sleep Efficiency [%] T2 | 0.68  (0.59, 0.74) | 0.64  (0.56, 0.75) | 0,560 | 0.579 | 0,057 |
| Sleep Latency [h] T1 | 0.39  (0.27, 0.54) | 0.56  (0.34, 0.79) | -1,910 | 0.056 | -0,193 |
| Sleep Latency [h] T2 | 0.36  (0.25, 0.58) | 0.43  (0.32, 0.72) | -1,230 | 0.221 | -0,113 |

| Table A13: Groupwise comparison of PA of patients with pre-existing metabolic disease and without pre-existing metabolic disease at timepoint T1 and T2. | | | | | |
| --- | --- | --- | --- | --- | --- |
|  | **Pre-existing metabolic disease** | |  |  |  |
|  | No  (n=36) | Yes  (n=62) |  |  |  |
|  | Mdn (IQR) | Mdn (IQR) | **z** | **p** | **r** |
| Inactive [h] T1 | 13.57  (12.49, 14.92) | 14.41  (13.04, 15.25) | -1.530 | 0.128 | -0.155 |
| Inactive [h] T2 | 13.81  (12.66, 15.05) | 13.85  (12.54, 15.11) | -0.059 | 0.956 | -0.006 |
| Light Activity [min] T1 | 49.50  (38.01, 68.73) | 44.36  (33.84, 74.10) | 0.722 | 0.472 | 0.073 |
| Light Activity [min] T2 | 42.00  (34.56, 55.56) | 51.32  (32.85, 78.51) | -1.290 | 0.198 | -0.130 |
| Moderate Activity [min] T1 | 57.43  (40.82, 79.35) | 47.75  (36.03, 67.96) | 1.290 | 0.198 | 0.130 |
| Moderate Activity [min] T2 | 53.97  (40.77, 80.54) | 49.82  (34.94, 69.46) | 1.300 | 0.196 | 0.131 |
| Vigorous Activity [min] T1 | 0.12  (0.04, 0.41) | 0.09  (0.04, 0.23) | 0.955 | 0.342 | 0.096 |
| Vigorous Activity [min] T2 | 0.13  (0.08, 0.42) | 0.07  (0.03, 0.18) | 2.300 | 0.022 | 0.232 |

| Table A14: Groupwise comparison of sleep parameters of patients with pre-existing metabolic disease and without pre-existing metabolic disease at timepoint T1 and T2. | | | | | |
| --- | --- | --- | --- | --- | --- |
|  | **Pre-existing metabolic disease** | |  |  |  |
|  | No  (n=34) | Yes  (n=64) |  |  |  |
|  | Mdn (IQR) | Mdn (IQR) | **z** | **p** | **r** |
| Time in Bed [h] T1 | 8.12  (7.51, 8.75) | 7.51  (7.00, 8.22) | 1.840 | 0.066 | 0.186 |
| Time in Bed [h] T2 | 8.01  (7.01, 8.34) | 7.86  (7.11, 8.47) | -0.131 | 0.899 | -0.013 |
| Sleepduration [h] T1 | 6.10  (4.90, 6.82) | 5.30  (4.12, 6.34) | 1.800 | 0.072 | 0.182 |
| Sleepduration [h] T2 | 5.40  (4.79, 6.76) | 5.60  (4.82, 6.22) | 0.321 | 0.751 | 0.032 |
| Wake after Sleeponset [h] T1 | 2.10  (1.48, 2.73) | 1.96  (1.57, 2.88) | -0.496 | 0.622 | -0.050 |
| Wake after Sleeponset [h] T2 | 1.90  (1.50, 2.56) | 2.08  (1.61, 2.69) | -1.040 | 0.301 | -0.105 |
| Sleep Regularity [%] T1 | 42.28  (35.02, 47.13) | 42.20  (33.07, 52.38) | -0.634 | 0.528 | -0.064 |
| Sleep Regularity [%] T2 | 41.16  (34.37, 50.59) | 44.60  (32.87, 51.72) | -0.239 | 0.814 | -0.024 |
| Sleep Efficiency [%] T1 | 0.69  (0.62, 0.77) | 0.67  (0.55, 0.75) | 1.110 | 0.268 | 0.112 |
| Sleep Efficiency [%] T2 | 0.68  (0.60, 0.77) | 0.66  (0.58, 0.74) | 0.713 | 0.478 | 0.072 |
| Sleep Latency [h] T1 | 0.36  (0.20, 0.56) | 0.44  (0.31, 0.57) | -1.650 | 0.100 | -0.167 |
| Sleep Latency [h] T2 | 0.35  (0.24, 0.49) | 0.37  (0.26, 0.61) | -0.877 | 0.383 | -0.081 |

| Table A15: Results of two-way ANOVA Sleep duration T1 ~ Sex + Age + Sex*Age | | | | | |
| --- | --- | --- | --- | --- | --- |
|  | Df | Sum Sq | Mean Sq | F | p |
| Sex | 1 | 12.789 | 12.789 | 7.421 | 0.008 |
| Age | 1 | 7.923 | 7.923 | 4.598 | 0.035 |
| Sex*Age | 1 | 7.596 | 7.596 | 4.408 | 0.038 |
| Residuals | 94 | 161.987 | 1.723 |  |  |
| R2 | 0.149 | | | | |
| R2 adj. | 0.122 | | | | |

| Table A16: Results of two-way ANOVA Sleep duration T2 ~ Metabolic disease + COVID-19 severity + Metabolic disease*COVID-19 severity | | | | | | |
| --- | --- | --- | --- | --- | --- | --- |
|  | Df | Sum Sq | Mean Sq | | F | p |
| Metabolic disease | 1 | 0.447 | 0.447 | | 0.330 | 0.567 |
| COVID-19 severity | 1 | 4.007 | 4.007 | | 2.955 | 0.089 |
| Metabolic disease*COVID-19 severity | 1 | 6.746 | 6.746 | | 4.975 | 0.028 |
| Residuals | 94 | 127.457 | 1.356 | |  |  |
| R2 | 0.081 |  |  | |  |  |
| R2 adj. | 0.051 |  |  |  | |  |

| Table A17: Results of two-way ANOVA Sleep regularity T1 ~ Metabolic disease + Cardiovascular disease + Metabolic disease*Cardiovascular disease | | | | | |
| --- | --- | --- | --- | --- | --- |
|  | Df | Sum Sq | Mean Sq | F | p |
| Metabolic disease | 1 | 70.161 | 70.161 | 0.385 | 0.536 |
| Cardiovascular disease | 1 | 52.036 | 52.036 | 0.286 | 0.594 |
| Metabolic disease*Cardiovascular disease | 1 | 780.626 | 780.626 | 4.284 | 0.041 |
| Residuals | 94 | 17128.223 | 182.215 |  |  |
| R2 | 0.050 | | | | |
| R2 adj. | 0.020 | | | | |

| Table A18: Results of two-way ANOVA Sleep regularity T2 ~ Sex + Cardiovascular disease + Sex*Cardiovascular disease | | | | | |
| --- | --- | --- | --- | --- | --- |
|  | Df | Sum Sq | Mean Sq | F | p |
| Sex | 1 | 146.332 | 146.332 | 0.830 | 0.365 |
| Cardiovascular disease | 1 | 73.280 | 73.280 | 0.416 | 0.521 |
| Sex*Cardiovascular disease | 1 | 1252.102 | 1252.102 | 7.100 | 0.009 |
| Residuals | 94 | 16578.002 | 176.362 |  |  |
| R2 | 0.082 | | | | |
| R2 adj. | 0.052 | | | | |

| Table A19: Results of two-way ANOVA Light PA T1 ~ Respiratory disease + Mental disorder + Respiratory disease*Mental disorder | | | | | |
| --- | --- | --- | --- | --- | --- |
|  | Df | Sum Sq | Mean Sq | F | p |
| Respiratory disease | 1 | 9037.905 | 9037.905 | 1.505 | 0.223 |
| Mental disorder | 1 | 696.342 | 696.342 | 0.116 | 0.734 |
| Respiratory disease*Mental disorder | 1 | 29116.893 | 29116.893 | 4.847 | 0.030 |
| Residuals | 94 | 564656.924 | 6006.989 |  |  |
| R2 | 0.064 | | | | |
| R2 adj. | 0.035 | | | | |

| Table A20: Results of two-way ANOVA Moderate PA T1 ~ Cardiovascular disease + Sex + Cardiovascular disease*Sex | | | | | |
| --- | --- | --- | --- | --- | --- |
|  | Df | Sum Sq | Mean Sq | F | p |
| Cardiovascular disease | 1 | 760.836 | 760.836 | 1.317 | 0.254 |
| Sex | 1 | 667.697 | 667.697 | 1.156 | 0.285 |
| Cardiovascular disease*Sex | 1 | 2360.059 | 2360.059 | 4.087 | 0.046 |
| Residuals | 94 | 54285.929 | 577.510 |  |  |
| R2 | 0.065 | | | | |
| R2 adj. | 0.035 | | | | |

| Table A21: Results of two-way ANOVA Moderate PA T1 ~ Metabolic disease + Sex + Metabolic disease*Sex | | | | | |
| --- | --- | --- | --- | --- | --- |
|  | Df | Sum Sq | Mean Sq | F | p |
| Metabolic disease | 1 | 1221.079 | 1221.079 | 2.201 | 0.141 |
| Sex | 1 | 412.108 | 412.108 | 0.743 | 0.391 |
| Metabolic disease*Sex | 1 | 4286.168 | 4286.168 | 7.725 | 0.007 |
| Residuals | 94 | 52155.166 | 554.842 |  |  |
| R2 | 0.102 | | | | |
| R2 adj. | 0.073 | | | | |

| Table A22: Results of two-way ANOVA Vigorous PA T2 ~ Metabolic disease + Sex + Metabolic disease*Sex | | | | | |
| --- | --- | --- | --- | --- | --- |
|  | Df | Sum Sq | Mean Sq | F | p |
| Metabolic disease | 1 | 15.925 | 15.925 | 2.582 | 0.111 |
| Sex | 1 | 10.154 | 10.154 | 1.646 | 0.203 |
| Metabolic disease*Sex | 1 | 26.604 | 26.604 | 4.314 | 0.041 |
| Residuals | 94 | 579.728 | 6.167 |  |  |
| R2 | 0.083 | | | | |
| R2 adj. | 0.054 | | | | |

| Table A22: Results of two-way ANOVA Vigorous PA T2 ~ Metabolic disease + Respiratory disease + Metabolic disease*Respiratory disease | | | | | |
| --- | --- | --- | --- | --- | --- |
|  | Df | Sum Sq | Mean Sq | F | p |
| Metabolic disease | 1 | 15.925 | 15.925 | 2.622 | 0.109 |
| Respiratory disease | 1 | 11.232 | 11.232 | 1.850 | 0.177 |
| Metabolic disease*Respiratory disease | 1 | 34.407 | 34.407 | 5.666 | 0.019 |
| Residuals | 94 | 570.846 | 6.073 |  |  |
| R2 | 0.097 | | | | |
| R2 adj. | 0.069 | | | | |
